# Influence of pelvic position on shoulder range of motion

**DOI:** 10.1186/s12891-025-08280-0

**Published:** 2025-01-17

**Authors:** Bishoy S. Lobbos, Mohamed M. M. Essa, Alaaeldin Khaireldin, Mohamed Y. Gamal El-Din, Phillips Rizkallah, Heba Allah Samy

**Affiliations:** 1 Lecturer of Orthopedic Physical Therapy, Faculty of Physical Therapy, Deraya University, Minia, Egypt; 2 Associate Professor of Biomechanics, Faculty of Physical Therapy, Sphinx University, Assuit, Egypt; 3 Department of Physical Therapy, Benha University Hospital, Benha University, Benha, Qalyubia Egypt; 4 Lecturer of Physical Therapy, Basic Science Department, Faculty of Physical Therapy, October University for Modern Sciences and Arts, 6th October, Giza, Egypt; 5 Faculty of Medicine, Sohag University, Sohag, Egypt; 6 Lecturer of Physical Therapy, Basic Science Department, Faculty of Physical Therapy, Suez University, Suez, Egypt

**Keywords:** Pelvis, Posture, Spine, Shoulder, Range, Motion

## Abstract

**Background:**

pelvis and shoulder are deeply integrated. They are connected by myofascial slings. The pelvic and spinal posture affects the position of the scapula and the activity of its muscles and affects acromio-humeral distance and so that affects shoulder movement. The aim of the study was to investigate the influence of pelvic position on the shoulder range of motion.

**Methods:**

The full active range of motion (flexion/extension, abduction, and external/internal rotation) of both shoulders was measured by digital goniometer for 33 normal adult subjects. Measurements were taken from a standing position in the following pelvic positions: 1- Neutral posture, 2- Evoked anterior and posterior pelvic tilt, 3- Evoked right and left pelvic rotation, 4- Evoked right and left lateral pelvic tilt. For every shoulder movement, One-Way ANOVA including Tukey post hoc test was used to compare between different positions.

**Results:**

Anterior pelvic tilt leads to a significant increase in flexion and a significant decrease in extension of both shoulders (P value was < 0.001). Posterior pelvic tilt leads to the opposite. Pelvic rotation leads to a significant decrease in shoulder flexion on the same side of rotation and shoulder extension on the opposite side of rotation (P value was < 0.001). Lateral pelvic tilt leads to a significant decrease in abduction on the same side of lateral tilt (P value was < 0.001).

**Conclusion:**

pelvic position affects shoulder range of motion.

## Background

The pelvis and shoulder are deeply integrated. Anatomically, the myofascial system connects the pelvis and shoulder. For example, posterior and anterior oblique slings connect the lumbopelvic region to the contralateral shoulder. The gluteus maximus extends into the thoracolumbar fascia through the posterior sling, crosses over to the contralateral latissimus dorsi, and terminates in the contralateral shoulder. The hip-lumbopelvic region is connected to the contralateral shoulder by the anterior oblique sling, which is made up of the hip adductors, transverse abdominis, internal and external oblique, anterior fascia of the trunk, and pectoralis major [1, 2].

Moreover, the shoulder’s kinematics can be influenced by these slings’ impairment. The contralateral humeral head is positioned incorrectly when there is lumbopelvic dysfunction; this can be caused by altered transmission of the myofascial force from the lumbopelvic region across the oblique sling muscle [3, 4].

In terms of mechanics, walking and mobility heavily depend on the integration of the shoulder and pelvis. Arm swing or counter rotation of the thoracic spine are used as adaptations to pelvic angular momentum [5].

Alterations in pelvic posture can impact the posture of the shoulder complex by causing changes in spinal posture. The lumbar lordosis is increased by anterior pelvic tilt and decreased by posterior pelvic tilt [6, 7]. Thus, thoracic kyphosis will be changed as a compensation [6]. Reduction in anterior pelvic tilt shifts the body’s center of mass posteriorly. As a result, the person reduces their extended trunk posture to maintain the center of mass in the best alignment with respect to the line of gravity in the anteroposterior plane [8].

The scapular position and electromyographic activity of the scapula’s muscles during shoulder movement are influenced by the posture of the spine [9]. Scapular elevation results from increased kyphosis of the thoracic spine or from a slouched posture. It reduces the scapula’s external, upward, and posterior tilting movements [10–12]. Scapular protraction and downward rotation can result from bending forward [13]. According to Murta et al. [8], alteration of scapular posture may change the length tension relationship of the scapular muscles, which in turn causes changes in their activity during shoulder movements. The middle and lower trapezius muscle’s activity can be elevated by a slouch posture [14].

Additionally, the activation of the scapular muscles is influenced by the pelvic position. Higher activation of the lower trapezius during arm movement and a less extended trunk posture are the outcomes of reducing anterior pelvic tilt [8]. To maintain this posture, the gluteus maximus contraction will cause tightness of the thoracolumbar fascia, causing destabilization of the scapula. As a result, the lower trapezius becomes more active [15]. Additionally, abdominal muscles contribute to the reduction of anterior pelvic tilt and lead to higher activity of the scapular muscles, particularly the lower trapezius [16].

Shoulder mobility is affected by variations in the acromio-humeral distance (AHD), which is caused by changes in spinal position. The AHD is increased by an upright position [17]. Conversely, a slouched posture or thoracic hyperkyphosis results in a decrease in the AHD [18]. Thus, assuming a slouched posture reduces shoulder abduction range and strength [10], and assuming an upright posture increases shoulder abduction and flexion range [19, 12].

The anterior scapular tilt and its decreased upward rotation result in decreased abduction and flexion. This is because of the blockage created by the acromion and resultant subacromial space narrowing. Additionally, the AHD is shortened by scapular protraction [10, 19] Since greater thoracic kyphosis alters the length-tension relationship of the shoulder girdle muscles, the reduced range may be related to the thoracic position itself. Thus, it may result in humeral head malalignment [19, 20].

This study aimed to assess if changing the pelvic position affects the shoulder range of motion or not.

## Methods

### Study design

This cross-sectional study aimed to investigate the influence of pelvic position on the shoulder range of motion. It was conducted at the Faculty of Physical Therapy, Deraya University.

### Participants

Thirty-three subjects were enrolled in this study. A subject was included in the study if their age ranged from 18 to 30 years and their body mass index (BMI) ranged from 18 to 24 kg/m^2^, subjects were excluded if they had shoulder, spinal and pelvic pain or disorders, leg length discrepancy, or any congenital or acquired deformation.

### Instruments

A digital goniometer (Baseline^®^ Digital Absolute Axis™ Goniometer 12-1027) was used to measure shoulder range of motion. It displays angles from 0° to 180° with an LCD screen and an angle precision of 0.5 °.

Concerning intra-rater reliability of the digital goniometer, the intraclass correlation coefficient (ICC) ranged from 0.635 to 0.97. The standard error of measurement (SEM) ranged from 1.93° to 7.6°, and the minimal detectable change (MDC) ranged from 5° to 11°. For inter-rater reliability, the ICC ranged from 0.477 to 0.996, the SEM ranged from 0.77° to 4.7°, and the MDC ranged from 2° to 9° [21–25].

### Procedures

**Fig. 1:**
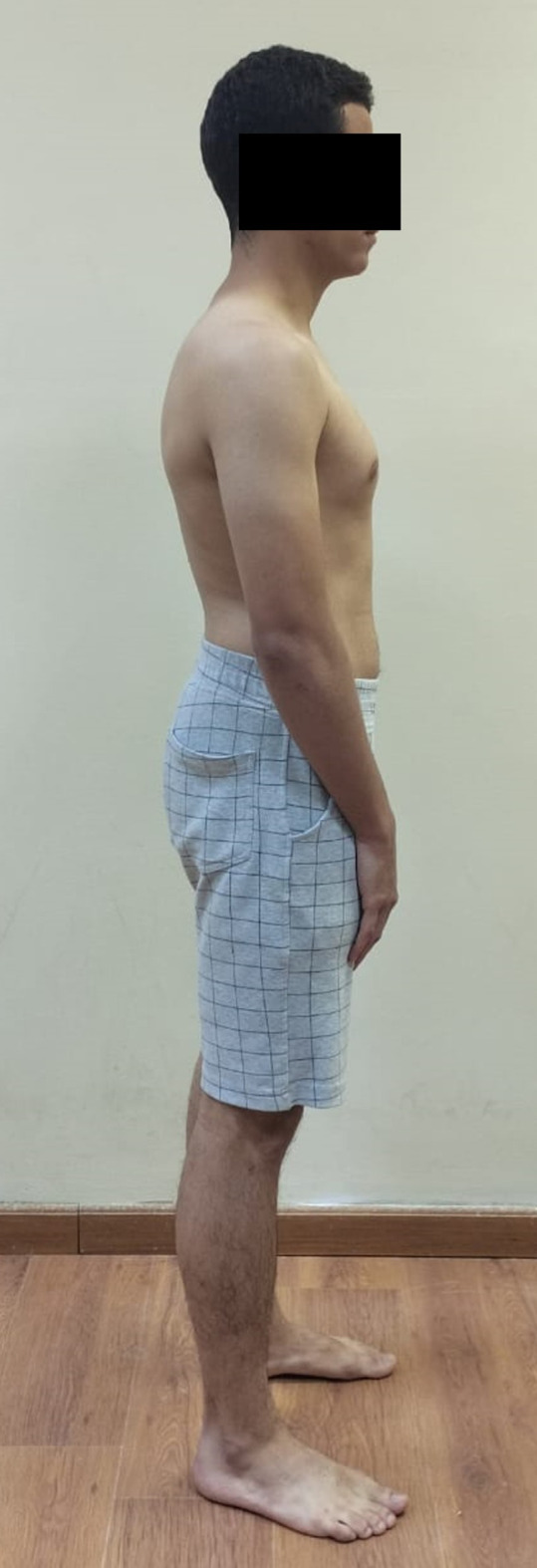
Neutral or natural pelvic posture

**Fig. 2:**
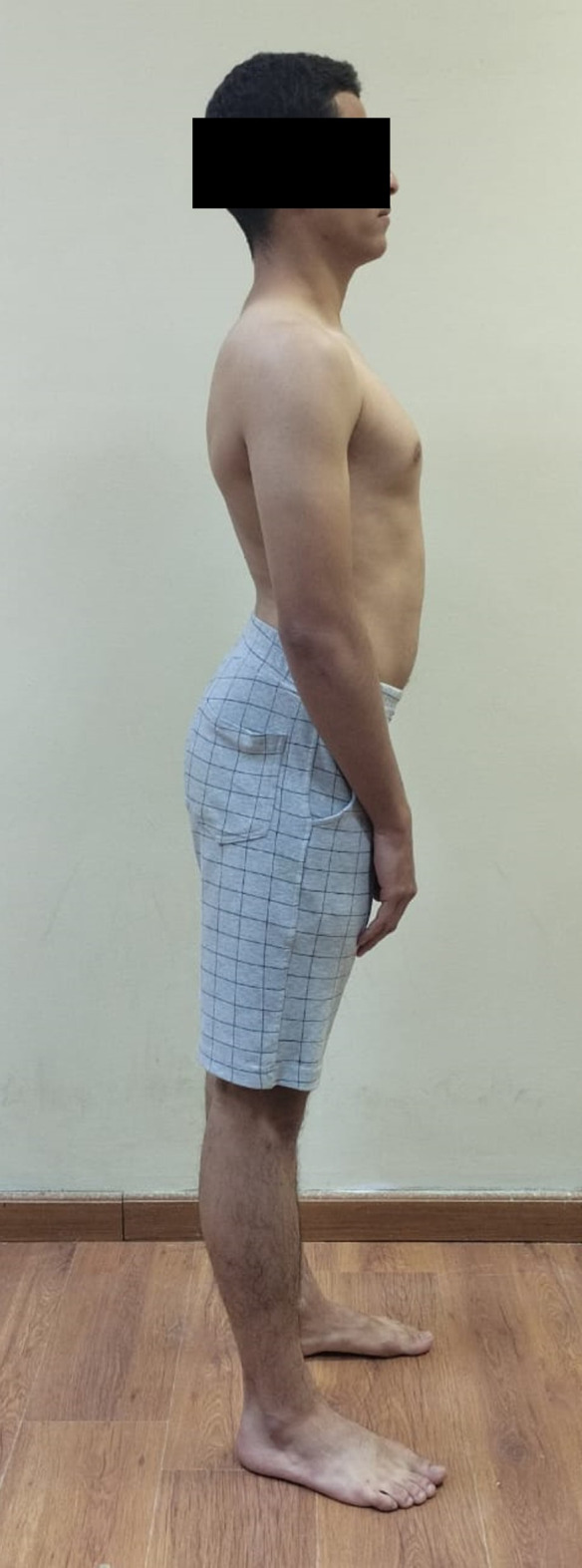
Evoked anterior pelvic tilt

**Fig. 3:**
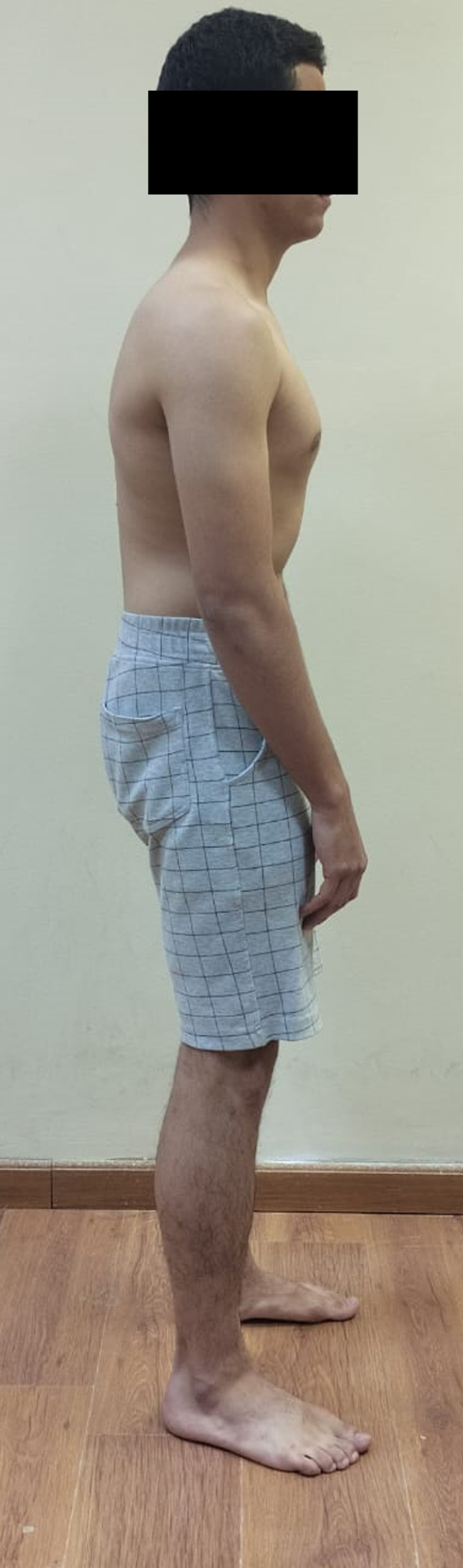
Evoked posterior pelvic tilt

**Fig. 4:**
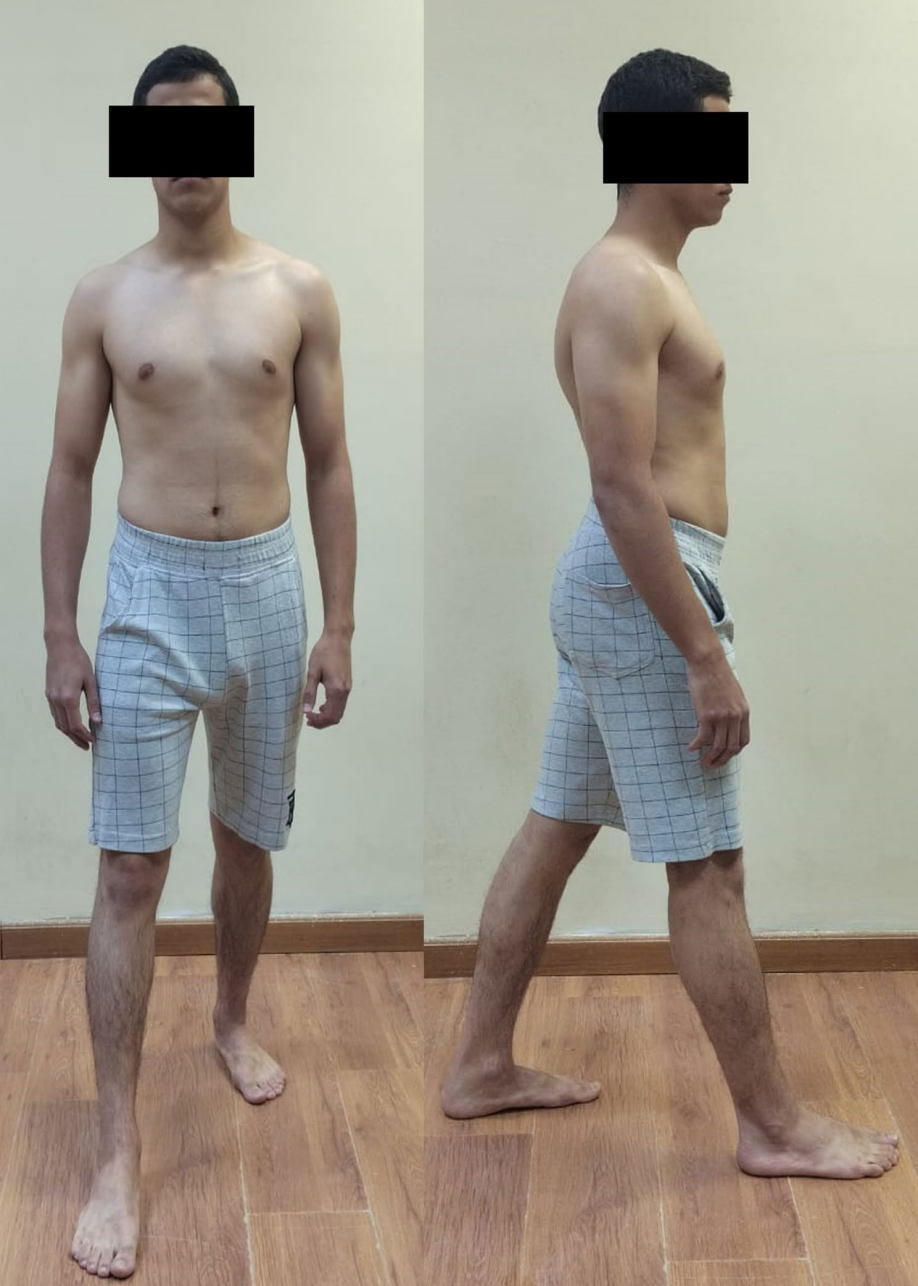
Evoked pelvic rotation; the right forward foot led to leftward pelvic rotation

**Fig. 5:**
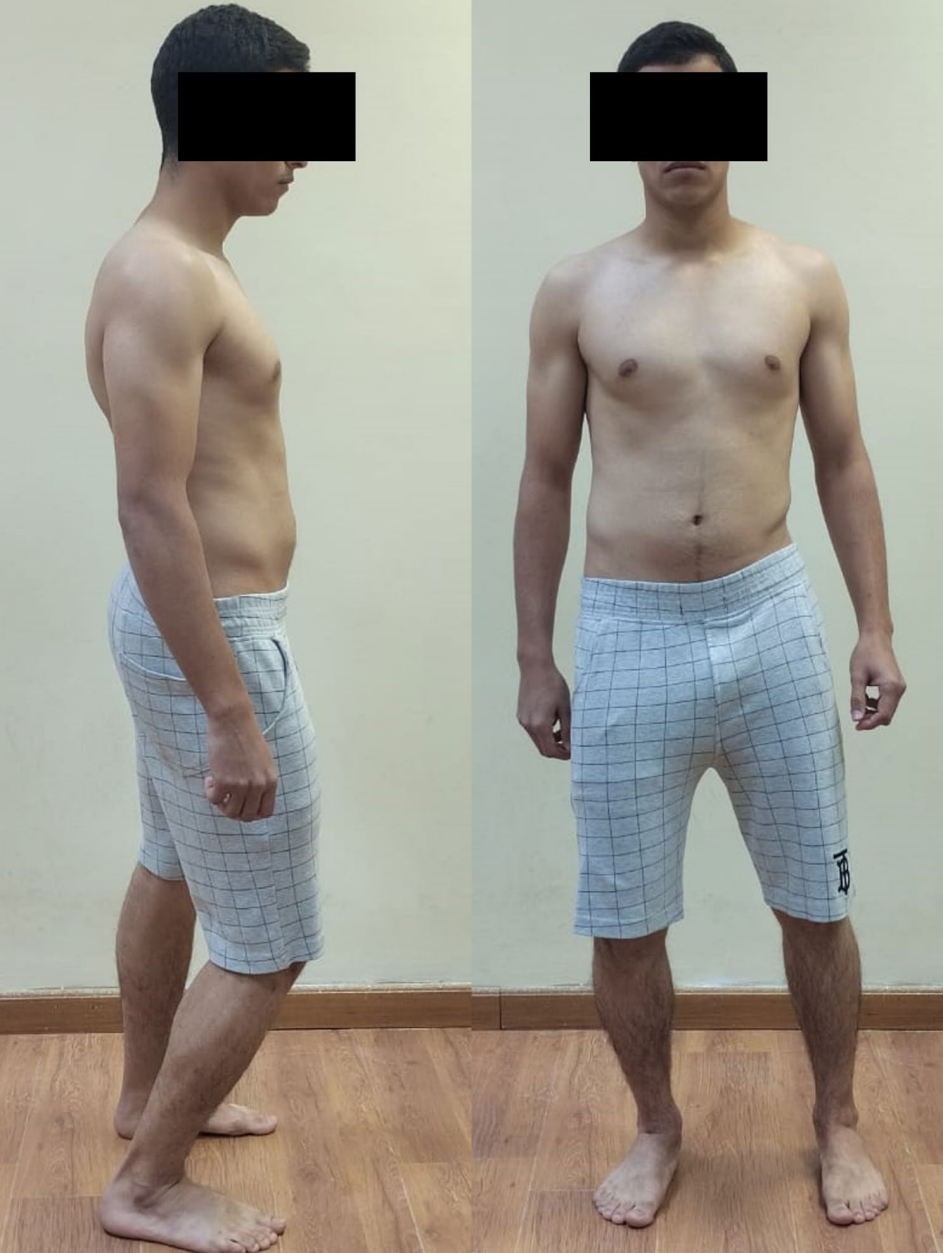
Evoked lateral pelvic tilt; subject stood with right knee slightly flexed, so that the pelvis tilted laterally to the right side

The full active range of motion of both shoulders was measured in all planes (flexion/extension, abduction, and external/internal rotation) from a standing position in the following pelvic positions:

1. Neutral or natural posture (Fig. 1).
2. Evoked anterior and posterior pelvic tilt. (subject actively performed anterior and posterior pelvic rotation) (Figs. 2 and 3).
3. Evoked right and left pelvic rotation (subject stood with one foot stepped forward, so that the pelvis was rotated to the opposite direction of the forward foot. i.e., the right forward foot led to leftward pelvic rotation.) (Fig. 4).
4. Evoked right and left lateral pelvic tilt (subject stood with one knee slightly flexed, so that the pelvis tilted laterally to the side of the flexed knee) (Fig. 5).

For flexion and extension measurement, the stationary arm of the goniometer was parallel to the lateral Midline of the thorax and the movable arm was placed on the lateral midline of the arm. For abduction, the stationary arm of the goniometer was parallel to the sternum and the movable arm was placed on the anterior midline of the arm. Concerning external and internal rotation, the shoulder was abducted 90 degrees, and the elbow was flexed 90 degrees. The stationary arm of the goniometer was parallel to the ground, with the axis on the olecranon process and the movable arm was placed on the ulnar border of the forearm.

### Statistical analysis

The sample size was calculated by G^*^Power3.1.9.7 with the statistical parameters; effect size f = 0.25, α error probability = 0.05, and power (1-β) = 0.80. Therefore, for every shoulder movement, each pelvic position was represented by 33 observations.

For statistical analysis, the Statistical Package for the Social Sciences (SPSS) version 26 was used to analyze the collected data. The Shapiro–Wilk test was used to assess data normality for the measured range, and the data were normally distributed. For each shoulder movement, a One-Way ANOVA including the Tukey post hoc test was used to compare between different pelvic positions.

## Results

Thirty-three male subjects were enrolled in the current study. Their mean age was 23.64 ± 2.32 years, their mean weight was 68.04 ± 5.4 kg, their mean height was 173 ± 4.16 cm, and their mean body mass index (BMI) was 21.65 ± 1.64 kg/m².

### Flexion

There was a statistically significant difference in flexion between different pelvic positions as determined by One-Way ANOVA. For the right shoulder, F(6,224) was 205.8 and the P-value was < 0.001. For the left shoulder, F(6,224) was 236.8 and the P-value was < 0.001. Compared to the natural pelvic position, the Tukey post hoc test showed that flexion was significantly increased in anterior pelvic tilt (P-value < 0.001), decreased in posterior pelvic tilt (P-value < 0.001) for both shoulders, decreased in right rotated pelvis (P-value < 0.001) for the right shoulder and in left rotated pelvis (P-value < 0.001) for the left shoulder. Conversely, there was no significant difference in flexion with left pelvic rotation for the right shoulder or right pelvic rotation for the left shoulder, or right and left tilted pelvis for both shoulders (Table 1).

### Extension

There was a statistically significant difference in extension between different pelvic positions as determined by One-Way ANOVA. For the right shoulder, F(6,224) was 158.9 and P-value was < 0.001. for the left shoulder, F(6,224) was 154.6 and P-value was < 0.001. In comparison to the natural pelvic position, using the Tukey post hoc test, extension was significantly decreased in anterior pelvic tilt and increased in posterior pelvic tilt for both shoulders, and decreased in left pelvic rotation for the right shoulder and right pelvic rotation for left shoulder (all P-values were < 0.001). However, there was no significant difference in the extension range with right pelvic rotation for the right shoulder, left pelvic rotation for the left shoulder, or right and left tilted pelvis for both shoulders (Table 1).

### Abduction

One-Way ANOVA showed a significant difference in abduction between different pelvic positions. For the right shoulder, F(6,224) was 72.17 and the P-value was < 0.001. for the left shoulder, F(6,224) was 127.3 and the P-value was < 0.001. Compared to the natural pelvic position, the Tukey post hoc test showed only a significant decrease of abduction range in right pelvic tilt for the right shoulder and left pelvic tilt for the left shoulder (P-value was < 0.001), while there was no significant difference in all other pelvic positions (Table 1).

### External and internal rotation

One-Way ANOVA showed no significant difference in external rotation between different pelvic positions. For the right shoulder, F(6,224) was 1.3 and the P-value was 0.255. For the left shoulder, F(6,224) was 0.45 and the P-value was 0.844. Similarly, in internal rotation, there was no significant difference between different pelvic positions. For the right shoulder, F(6,224) was 0.984 and the P-value was 0.437. For the left shoulder, F(6,224) was 1.006 and the P-value was 0.423 (Table 1).

Table 1 shows the mean values of shoulder range of motion and the P-value for differences in shoulder range for every pelvic position when compared to the natural position using the Tukey post hoc test.

**Table 1:** Mean and P values for differences in shoulder range for every pelvic position compared to neutral

Pelvic Position	Flexion				extension				Abduction				External Rotation				Internal Rotation			
	Right		Left		Right		Left		Right		Left		Right		Left		Right		Left	
	Mean ±SD	*p*-value	Mean ±SD	*p*-value	Mean ±SD	*p*-value	Mean ±SD	*p*-value	Mean ±SD	*p*-value	Mean ±SD	*p*-value	Mean ±SD	*p*-value	Mean ±SD	*p*-value	Mean ±SD	*p*-value	Mean ±SD	*p*-value
Natural pelvic position	175.8 ± 2.1		176.66 ± 2.6		54.73 ± 2.9		54.43 ± 2.9		176.4 ± 1.35		176.66 ± 1.3		88.9 ± 1.24		88.23 ± 1.8		72.13 ± 3.2		72.93 ± 3.2	
Anterior Pelvic Tilt	180.36 ± 2.2	< 0.001*	181.56 ± 1.3	<0.001*	45.23 ± 1.8	<0.001*	46.53 ± 2	< 0.001*	176.86 ± 1.2	0.962	176.76 ± 1.27	1	89.03 ± 1	0.999	88.56 ± 1.67	0.967	70.83 ± 3.19	0.963	70.83 ± 3.03	0.678
Posterior Pelvic Tilt	165.36 ± 2	< 0.001*	165.26 ± 2.9	<0.001*	62.43 ± 2	<0.001*	63.53 ± 2.2	< 0.001*	176.66 ± 1.24	0.998	176.56 ± 1.35	1	88.53 ± 0.86	0.804	88.03 ± 1.37	0.998	72.23 ± 3.27	1	72.93 ± 3.2	1
Right Pelvic Rotation	165.96 ± 2.2	< 0.001*	178 ± 1.6	0.137	56.13 ± 2.9	0.349	46.43 ± 1.6	< 0.001*	176.76 ± 1.57	0.989	175.86 ± 1.8	0.494	88.6 ± 0.89	0.915	88.23 ± 1	1	70.73 ± 3.09	0.947	71.03 ± 3.04	0.772
Left Pelvic Rotation	176.36 ± 2.4	0.951	166.36 ± 2.4	<0.001*	46.73 ± 2	<0.001*	55.53 ± 3.25	0.654	175.86 ± 1.38	0.928	176.36 ± 1.4	0.992	88.5 ± 0.9	0.732	88.26 ± 1.17	1	72.03 ± 3.1	1	71.83 ± 2.9	0.980
Right Pelvic Tilt	175.86 ± 2.3	1	176.36 ± 2.3	0.99	55.43 2.9	0.940	55.13 ± 2.7	0.943	169 ± 3.7	< 0.001*	177.9 ± 0.95	0.062	88.6 ± 1.13	0.915	88.3 ± 1.1	1	73.5 ± 4	0.953	72.63 ± 2.96	1
Left Pelvic Tilt	175.7 ± 1.9	1	176.66 ± 1.7	1	55.33 ± 2.96	0.971	54.73 ± 2.9	0.999	177.23 ± 1.16	0.6	167.86 ± 2.7	< 0.001*	88.5 ± 1	0.732	88.1 ± 1.29	1	72.43 ± 2.9	1	72.93 ± 3.03	1

SD: Standard Deviation. *P* < 0.05 = significant *

## Discussion

The aim of this study was to investigate how pelvic position affects shoulder range of motion. Earlier research examined the connection between the scapula and the thoracic spine, two nearby structures, and the shoulder’s range of motion. While some studies focused on the impact of thoracic spine posture on scapular movement [10–13], others examined the connection between spinal posture and acromiohumeral distance (AHD) [17, 18]. Moreover, other research had determined the connection between shoulder range of motion and thoracic spine position [10, 12, 19].

Regarding flexion and extension, the current study showed that an anterior pelvic tilt significantly increases flexion and decreases extension of both shoulders. A posterior pelvic tilt does the opposite. The increased flexion with anterior pelvic tilt may be due to the anterior translation of the body center of mass (associated with anterior pelvic tilt) so that the individual extends the trunk to maintain the center of mass in good alignment in relation to the line of gravity and within the base of stability. This extension or upright posture allows the scapula to achieve a good position and increase the AHD [17] and thus increase flexion. The opposite occurs with posterior pelvic tilt, the center of mass moves posteriorly due to the posterior tilt, and the individual decreases the trunk extension [8]. This decreased extension or slouch posture decreases upward rotation, posterior tilting, and external rotation of the scapula [10–12] and decreases the AHD [18] and thus decrease flexion.

The decreased extension of both shoulders with anterior pelvic tilt may be due to increased tension of the anterior myofascial sling during an upright or extended posture. This tension is transmitted from the pelvic area to the contralateral shoulder [3, 4]. While the increased extension with posterior pelvic tilt may be due to decreased tension or relaxation of this sling during a less extended or slouch posture.

Pelvic rotation was found to cause a significant decrease in shoulder flexion on the same side of rotation and a decrease in shoulder extension on the opposite side of rotation. This change can be justified by an alteration in tension of both anterior and posterior oblique slings during pelvic rotation. Also, it can be related to trunk rotation itself as unilateral shoulder movement requires ipsilateral upper thoracic rotation and lateral flexion with extension [26, 27]. Lateral pelvic tilt didn’t significantly affect shoulder flexion or extension, which means that flexion and extension of the shoulder are affected by sagittal movement and rotation of the trunk more than frontal trunk movement.

Concerning shoulder abduction, the only significant difference was associated with lateral pelvic tilt. Lateral pelvic tilt leads to a significant decrease in abduction on the same side of the lateral tilt. This may be attributed to a change in spinal curvature and myofascial tension caused by lateral pelvic tilt.

Regarding external and internal rotation of the shoulder, pelvic position doesn’t significantly affect shoulder rotation. Although an anterior pelvic tilt produced an increase in external rotation of both shoulders and a posterior pelvic tilt produced a decrease in external rotation, these differences were not statistically significant. Additionally, anterior pelvic tilt produced a decrease in internal rotation, but this was not statistically significant. This finding means that shoulder rotation isn’t affected significantly by spinal and pelvic position.

The findings of this current study revealed that changes in pelvic position significantly affect sagittal shoulder movements (flexion and extension) and, to a lesser extent, shoulder abduction, with no impact on shoulder rotation. The current study pointed out the integrated relationship between pelvic position and shoulder range of movement. So that, patients with shoulder movement limitations should be examined and treated using an integrated model that’s include pelvis and spine.

### Limitations

This study was conducted using evoked pelvic positions, not real and permanent postures where soft tissue adaptation and structural accommodation occur. The measurements were taken from a standing position only; changing the measurement position to sitting or supine could alter soft tissue tension and muscle activity, thus it may affect results. The magnitude of change in different evoked positions of the pelvis from the natural position was not measured, preventing a statistical correlation with changes in shoulder range of motion. Regarding measurements, the testing of intra-rater and the inter-rater reliability of the examiners wasn’t performed. Additionally, the measurement error wasn’t specified.

Further studies are required to examine shoulder range of motion in permanent abnormal or mechanically impaired pelvic postures, or on opposite way to examine pelvic posture in problematic shoulder.

## Conclusion

Pelvic position influences sagittal shoulder movement (flexion and extension), and to a lesser extent shoulder abduction with no effect on shoulder rotation (external and internal rotation). An anterior pelvic tilt significantly increases flexion and decreases extension of both shoulders while a posterior pelvic tilt does the opposite. Pelvic rotation significantly decreases flexion on the same side of rotation and decreases extension on the contralateral side. Lateral pelvic tilt leads to a significant decrease in abduction on the same side of the lateral tilt.

## Data Availability

Table 1 contains the datasets necessary for interpretation, any other datasets are available from the corresponding author on reasonable request.

## Abbreviations

AHD: Acromio-Humeral Distance
BMI: Body Mass Index
ICC: Intraclass Correlation Coefficient
MDC: Minimal Detectable Change
SEM: Standard Error of Measurement
